# Minimally invasive surgery as a new clinical standard for bone anchored hearing implants—real-world data from 10 years of follow-up and 228 surgeries

**DOI:** 10.3389/fsurg.2023.1209927

**Published:** 2023-07-03

**Authors:** Leonardo Di Santana Cruz, Fabiana Danieli, Maria Åberg Håkansson, Martin Lars Johansson, Francine Raquel dos Santos, Ana Claudia Mirândola Barbosa Reis, Miguel Angelo Hyppolito

**Affiliations:** ^1^ Department of Ophthalmology, Otorhinolaryngology, Head and Neck Surgery, Ribeirão Preto Medical School, University of São Paulo, Ribeirão Preto, Brazil; ^2^ Department of Health Sciences, RCS, Ribeirão Preto Medical School, University of São Paulo, Ribeirão Preto, Brazil; ^3^ Clinical Department, Oticon Medical, São Paulo, Brazil; ^4^ Clinical Department, Oticon Medical, Askim, Sweden; ^5^ Department of Biomaterials, Institute of Clinical Sciences, Sahlgrenska Academy, University of Gothenburg, Gothenburg, Sweden; ^6^ Research and Technology Department, Oticon Medical, Askim, Sweden

**Keywords:** bone conduction implant, minimally invasive surgery, osseointegrated hearing aid, real-world data, bone-anchored hearing system (BAHS), ponto

## Abstract

**Purpose:**

To explore the clinical practice development of different surgical techniques when installing bone-anchored hearing implants and their associated trends in outcomes.

**Design:**

Retrospective study of 228 bone-anchored hearing implants in 200 patients, performed over a 10-year period between 2012 and 2022 in a referral hospital.

**Method:**

Real-world data of demography, etiology, surgical setup, complications, and audiological outcomes were collected. Eligibility criteria from clinical practice were applied.

**Results:**

The minimally invasive technique is associated with shorter surgery duration, 20 vs. 44 min as compared to a linear incision technique. The minimally invasive technique was also associated with a lower occurrence of complications when compared to linear incision techniques (intraoperative; 1.8% vs. 4.9%, postoperative; 49% vs. 66%). Most differences were seen in complications relating to skin and wound healing.

**Conclusion:**

Adoption of a minimally invasive surgical technique for the installations of bone-anchored hearing implants can reduce surgical complexity without compromising safety aspects or clinical benefits.

## 1. Introduction

The bone-anchored hearing systems (BAHS), first introduced 1977 (1), have developed extensively in recent years. In addition to improvements in implant design and sound processing, there have been advances in the surgical technique used for installing the percutaneous BAHS implant. The original techniques involved extensive skin thinning (2) and a decade ago, the linear incision with tissue preservation techniques were introduced, where no or limited subcutaneous tissue is removed around the implant (3). This development has led to shorter surgical time, faster healing, and a more cosmetically pleasing results with less sever soft tissue complications (4, 5). More recently, minimally invasive techniques aiming to further reduce the impact on the soft tissue have been developed and introduced into clinical practice (6–8).

The minimally invasive Ponto surgery (MIPS) technique was first described in 2017 by Johansson et al., as a refinement of the tissue preservation techniques using a 5-mm punch incision (9). The method has been technically evaluated (10) and verified in randomized controlled trials for both short and long term benefits (6, 11). Further improvements were introduced by the MONO procedure, where the osteotomy is created using a one-step drill procedure, in contrast to the linear incision and MIPS techniques where a 3-step drilling procedure is employed (12, 13). Due to the single drill step approach, the MONO procedure is intended for 4 mm implants and adult patients only.

Several researchers have reported comparisons between linear incision and minimally invasive surgical techniques for bone-anchored hearing implant installation; however, these studies are often limited by small cohort sizes (14–18). The present study reports an extensive cohort of 228 bone-anchored hearing implants in 200 patients, representing unadjusted real-world data from introducing minimally invasive surgical techniques for bone-anchored hearing implant installations as the new clinical standard.

## 2. Materials and methods

This study is a retrospective study of 228 bone-anchored hearing implants in 200 patients, performed over a 10-year period between 2012 and 2022 in a university hospital in São Paulo, Brazil. The study was approved by the institutional Research Ethics Committee [i.e., Clinical Hospital of the Ribeirão Preto Medical School, University of São Paulo” – HCFMRP research Ethics Committee (CEP/HCFMRP)] under protocol n. 01508318.5.0000.5440. The setting of the study was the referral hospital from the Ribeirão Preto Medical School, University of São Paulo, involving approximately 35 otologic surgeons, fellows, and other trainees during this period.

**Figure 1:**
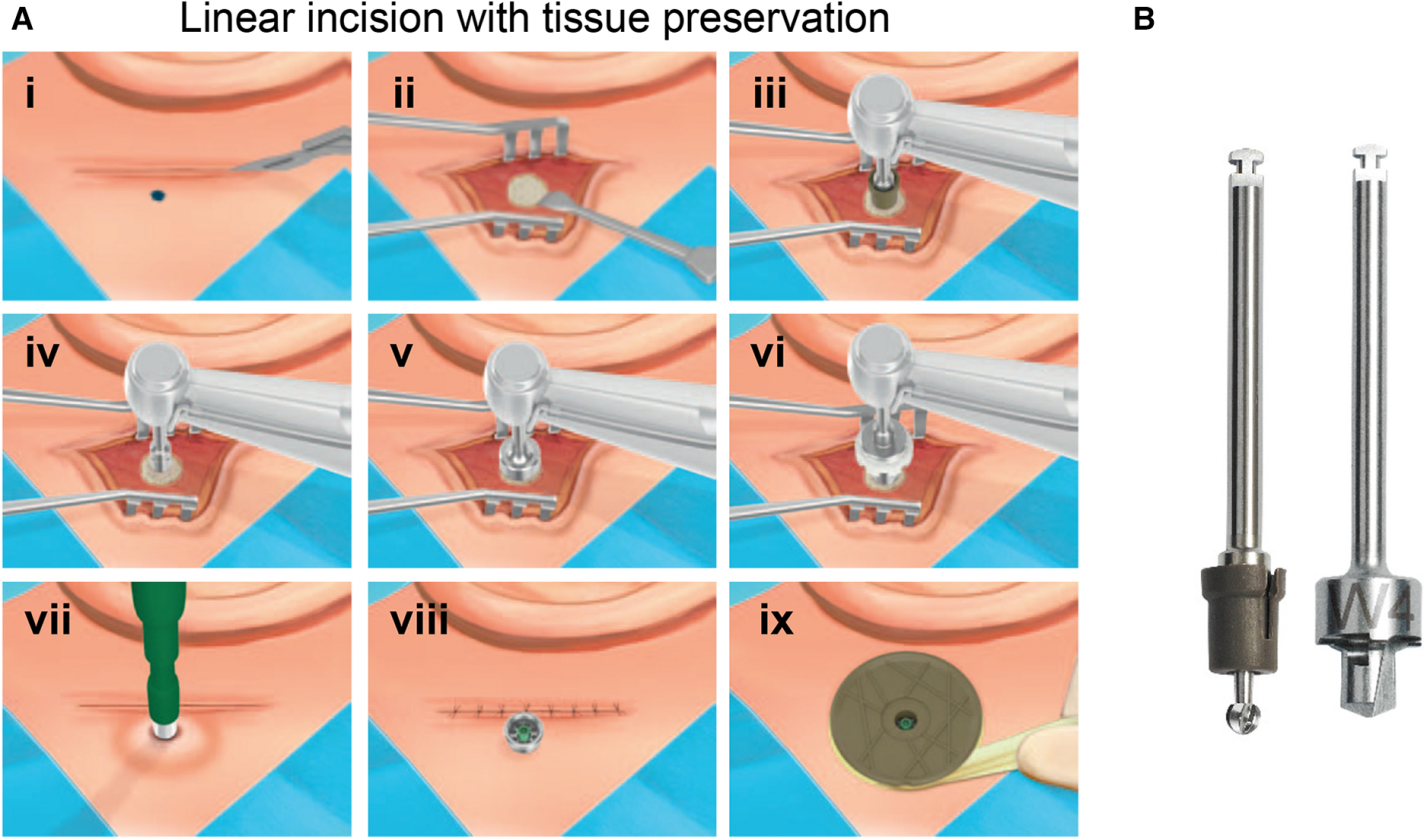
(**A**) Surgical steps for the linear incision with tissue preservation technique for installing BAHS. The following steps are executed; (i) A 2 cm–4 cm long linear incision is made and (ii) the periosteum around the surgical site is removed. (iii) The bone is penetrated with the Guide drill down to a depth of 4 mm. (iv) If the bone thickness is sufficient, the spacer is removed from the Guide drill to prepare for a 4-mm implant. (v) The hole is widened with the countersink. (vi) The implant installed (vi). (vii) A hole in the skin over the abutment is made using a Ø 5 mm biopsy punch. (viii) The skin is eased over the abutment and the incision closed. (ix) Finally, a healing cap is snapped onto the abutment and dressing is applied. (**B**) Drill system for the linear incision technique. Guide drill with removable spacer (left), countersink, 4 mm (right). Images reproduced by kind permission of Oticon Medical AB ©.

**Figure 2:**
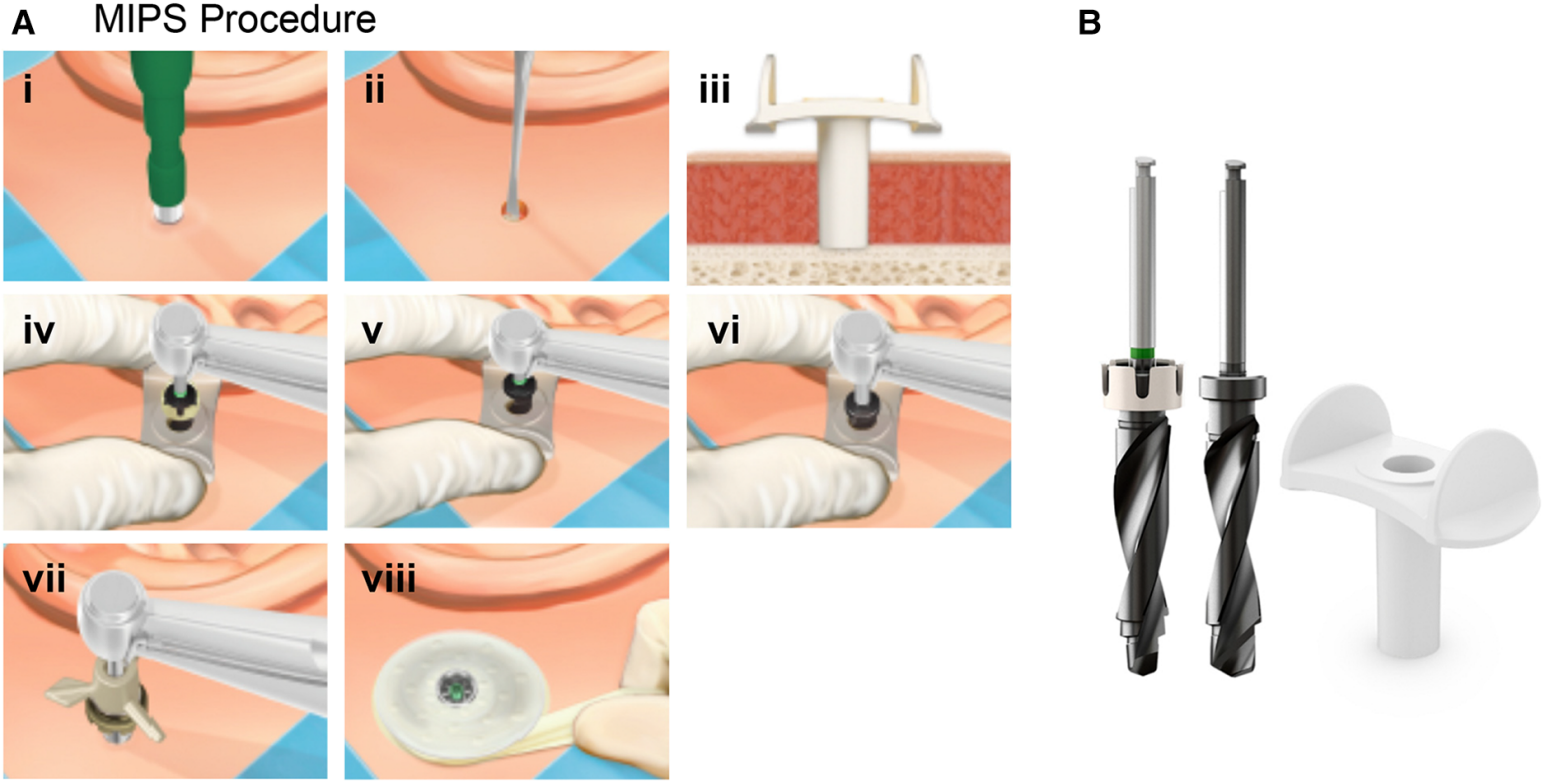
(**A**) Surgical steps for the minimally invasive ponto surgery (MIPS). The steps for the MIPS procedure are: (i) At the chosen site a circular incision is made using a 4 or 5-mm biopsy punch. (ii) The periosteum is removed around the surgical site. (iii) The cannula is inserted. (iv) Initial guide drilling is performed through the cannula. (v) If the bone thickness is sufficient, the spacer is removed from the Guide drill to prepare for a 4-mm implant. (vi) The hole is widened with the widening drill. (vii) The cannula is removed, and the implant installation is performed through the circular incision. (viii) Finally, a soft healing cap is attached to the abutment and dressing is applied. (**B**) The MIPS drill system: Guide drill with removable spacer (left), Widening drill, 4 mm (middle) and Cannula (left). Images reproduced by kind permission of Oticon Medical AB ©.

**Figure 3:**
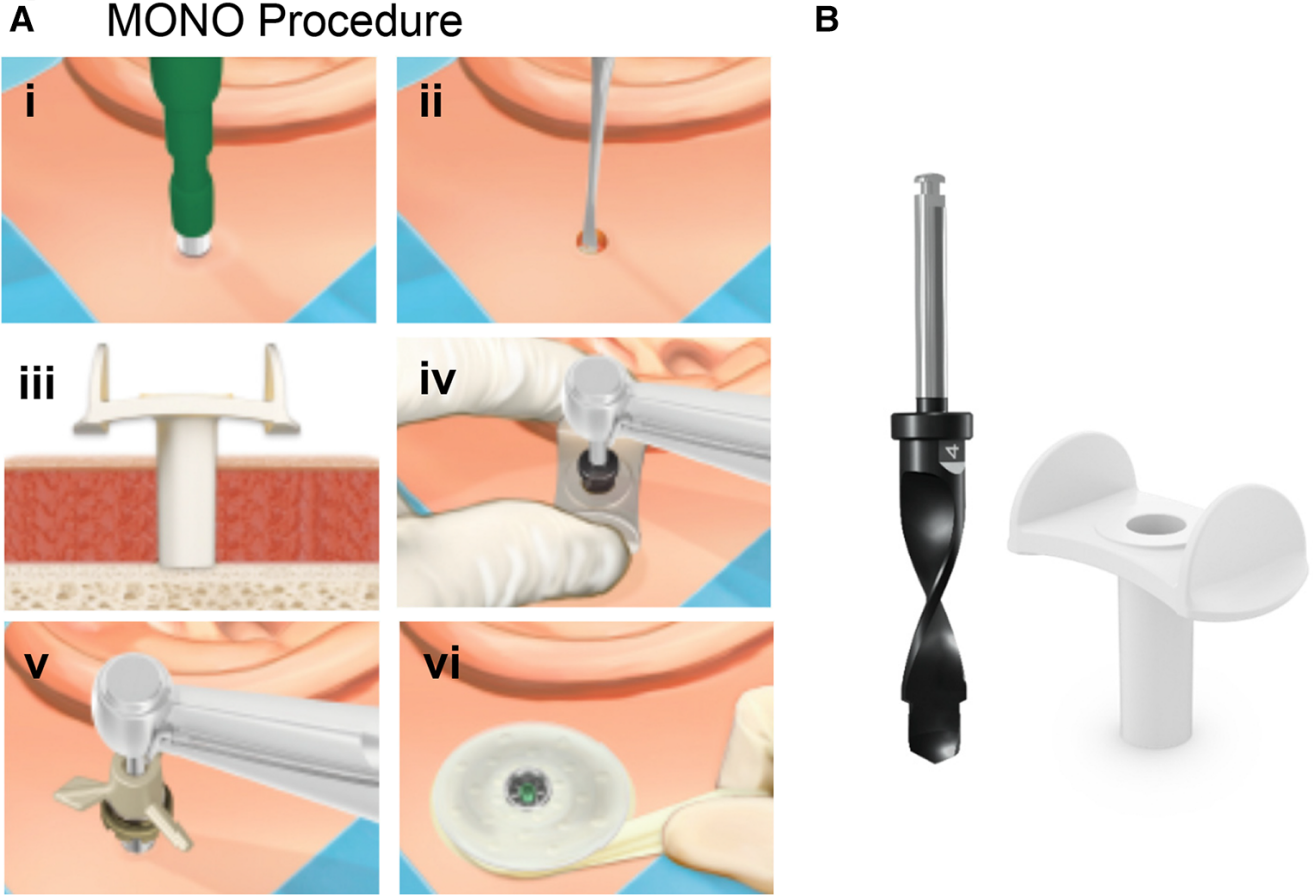
(**A**) Surgical steps for the MONO. The steps for the MONO procedure are: (i) At the chosen site a circular incision is made using a 4 or 5-mm biopsy punch. (ii) The periosteum is removed around the surgical site. (iii) The cannula is inserted. (iv) The final osteotomy is generated in one step using the MONO drill. (vii) The cannula is removed, and the implant installation is performed through the circular incision. (viii) Finally, a soft healing cap is attached to the abutment and dressing is applied. (**B**) The MONO drill system: MONO drill (left) and Cannula (left). Images reproduced by kind permission of Oticon Medical AB ©.

The first BAHS surgery was performed in this service in 2010, with the use of a single vertical incision technique with skin thinning. In 2012, the use of Ponto device (Oticon Medical AB, Sweden) with different abutment lengths allowed the use of the linear incision surgical technique without skin thinning and, due to the improved cosmetic and peri-implant infection results, this procedure was routinely adopted for all types of BAHS surgery (Figure 1). The first surgical procedures employing minimally invasive techniques (without the linear incision) were performed in the service in 2017. With the MIPS technique, bone-anchored hearing implant surgeries started to be routinely performed on an ambulatory basis under local anesthesia (2% lidocaine with 1:200,000 epinephrine), in most patients (Figure 2). The one-step MONO drilling procedure was first adopted by the service in 2022, and since then, it has been employed in most bone-anchored hearing implant surgeries of patients 18 years and older (Figure 3).

The aim of the study was to retrospectively compare the outcomes following BAHS implantation using different surgical techniques. To reflect a real-world life setting, eligibility criteria from clinical practice were applied and surgical methods studied followed the clinical practice development of the institution over a 10-years period. Data collected included information about demography and background data (age, gender, hearing loss etiology), surgical technique and setup (implanted ear/s, procedure, duration, anesthesia, implant type/length), and complications and follow-up data [intra/postoperative complications, Holgers score (19, 20), scarring, pain, numbness, implant loss, revision surgery, abutment change]. Additionally, baseline and follow-up audiological measurements were collected including audiological diagnosis, pure tone average from frequencies of 500, 1,000, 2,000, 3,000 and 4,000 Hz, and speech recognition by means of monosyllabic recognition (16). All data points were derived from medical records and represent standard follow-up measures used in clinical practice. For example, surgery duration represents time between first incision/punch and last suture, and the five-graded Holgers score was used to assess soft-tissue status. The typical follow-up visit after surgery is at 2 weeks, 1 month, and 3 months with the main purpose of verifying implant stability and complications related to the surgery site (e.g., skin reaction, pain, numbness). Then, annual audiological follow-up runs continuously. To minimize the number of missing values during follow-up, occurrences were registered irrespectively of timepoint of assessment and data is presented with full transparency of available sample size (in comparison to total sample size) in the tables.

### 2.1. Statistical method

Descriptive results are presented as *n* (%) for categorical variables and as the mean (SD) and median (min; max) for continuous variables. For comparisons between groups, a *t*-test was used for continuous variables, Fisher's Exact test (lowest 1-sided *p*-value multiplied by 2) was used for dichotomous variables, and the Mantel–Haenszel *χ*^2^ test was used for ordered categorical variables. For pairwise comparison between groups Fisher's Exact test (2-sided) was used for dichotomous variables and the Mantel–Haenszel *χ*^2^ test was used for ordered categorical variables. All statistical analyses were performed using SAS as statistical software and the level of significance was set at 5%.

In this study, most data reflect unilateral procedures and although some patients will reappear twice due to revision and/or bilateral procedures, all collected data follow the implant level and will therefore be presented as such. For clarity, demographic and background data are presented on subject level.

## 3. Results

This study included a total of 228 bone-anchored hearing implants in 200 patients, performed over a 10-year period between 2012 and 2022. A minimally invasive technique was used in 73% (*n* = 167) of the cases, divided between MIPS (65%, *n* = 149) and MONO (8%, *n* = 18), and the linear technique was performed in 27% (*n* = 61) of the cases. The total representation of men and women was 41% and 59%, respectively, and the mean age at surgery was 44 years, ranging between 6 and 77 years. Full disclosure of demographics is presented in Table 1.

**Table 1:** Demography, presented per subject.

	MIPS (*n* = 133, 66.5%)	MONO (*n* = 18, 9.0%)	Minimal invasive (MIPS + MONO) (*n* = 151, 75.5%)	Linear incision (*n* = 49, 24.5%)	Total (*n* = 200)
Age at surgery (years)					
Mean (SD, range)	45.5 (17.4)	49.9 (15.2)	46.1 (17.2)	38.3 (21.1)	44.1 (18.5)
Median (Min; Max)	47.0 (6; 77)	53.5 (22; 68)	(6; 77)	40.0 (6; 73)	46.0 (6; 77)
Age groups, *n* (%)					
≤12 years	7 (5.3%)	0 (0.0%)	7 (4.6%)	9 (18.4%)	16 (8.0%)
13–69 years	115 (86.5%)	18 (100.0%)	133 (88.1%)	38 (77.6%)	171 (85.5%)
≥70 years	11 (8.3%)	0 (0.0%)	11 (7.3%)	2 (4.1%)	13 (6.5%)
Gender, *n* (%)					
Female	86 (64.7%)	8 (44.4%)	94 (63.3%)	24 (49.0%)	118 (59.0%)
Male	47 (35.3%)	10 (55.6%)	57 (37.7%)	25 (51.0%)	82 (41.0%)
Primary hearing loss etiology, *n* (%)	(*n* = 132)	(*n* = 18)	(*n* = 150)	(*n* = 49)	(*n* = 199)
Mastoidectomy cavity	74 (56.1%)	11 (61.1%)	85 (56.7%)	27 (55.1%)	112 (56.3%)
Chronic otitis media	18 (13.6%)	3 (16.7%)	21 (13.9%)	6 (12.2%)	27 (13.6%)
External auditory canal atresia	3 (2.3%)	0 (0.0%)	3 (2.0%)	2 (4.1%)	5 (2.5%)
Otosclerosis	2 (1.5%)	2 (11.1%)	4 (2.6%)	1 (2.1%)	5 (2.5%)
External auditory canal stenosis	4 (3.0%)	0 (0.0%)	4 (2.6%)	2 (4.1%)	6 (3.0%)
Middle Ear Cholesteatoma	3 (2.3%)	0 (0.0%)	3 (2.0%)	2 (4.1%)	5 (2.5%)
Microtia	0 (0.0%)	0 (0.0%)	0 (0.0%)	1 (2.0%)	1 (0.5%)
Other*	5 (3.8%)	0 (0.0%)	5 (3.3%)	2 (4.1%)	7 (3.5%)
Not defined	23 (12.2%)	2 (11.1%)	25 (16.6%)	6 (12.2%)	31 (15.6%)
Unilateral/Bilateral, *n* (%)				(*n* = 50**)	(*n* = 201**)
Unilateral	126 (94.7%)	18 (100.0%)	144 (95.4%)	46 (92.0%)	190 (94.5%)
Bilateral	7 (5.3%)	0 (0.0%)	7 (4.6%)	4 (8.0%)	11 (5.5%)

* Single/few observations of acoustic neuroma, middle ear fibrous dysplasia, middle ear hemangionoma, and external ear choleasteatoma.

** One patient had unilateral and bilateral procedure done at different occasions.

There was a high general variability in the demographic variables in the total population, and some skewness was observed between the groups based on surgical technique, i.e., a higher mean age at surgery was observed in the minimally invasive group than in the linear incision technique group. The majority of hearing loss etiology reported was mastoidectomy cavity (56%). This was similar between the two surgical techniques. Approximately 13% of cases were due to chronic otitis media and the remaining etiologies were less common and varied as disclosed in Table 1.

The vast majority of surgeries were one-stage procedures (99%) and performed under local anesthetics only (97%). The low number of cases using general anesthesia (*n* = 7) compromises trend analysis related to age and/or complications rate, but it was observed that two out of the seven cases with reported general anesthesia were children (age = 6 years). The surgeries were performed unilaterally in 95% of the cases, with a relatively even distribution between the left (53%) and right (47%) ears. This was observed irrespective of surgical technique (Tables 1, 2). The surgical procedure time was significantly shorter in the minimally invasive group than in the linear incision group, with mean times of 19.5 min (SD = 9.6) and 43.9 (SD = 22.2), respectively (*p* < 0.0001). There was no statistically significant difference in surgery time between MIPS and MONO. The primary implant represented by the data are a 4 mm implant (94%) (Oticon Medical Askim, Sweden), used together with an abutment length of 9 or 12 mm (Table 2).

**Table 2:** Surgical set-up and characteristics, presented per implant.

	MIPS (*n* = 149, 65.4%)	MONO (*n* = 18, 7.9%)	Minimal invasive (MIPS + MONO) (*n* = 167, 73.2%)	Linear incision (*n* = 61, 26.8%)	Total (*n* = 228)
Ear, *n* (%)					
Left	81 (54.4%)	7 (38.9%)	88 (52.7%)	32 (52.5%)	120 (52.6%)
Right	68 (45.6%)	11 (61.1%)	79 (47.3%)	29 (47.5%)	108 (47.4%)
Procedure type, *n* (%)					
One-stage	149 (100.0%)	18 (100.0%)	167 (100.0%)	59 (96.7%)	226 (99.1%)
Two-stage	0 (0.0%)	0 (0.0%)	0 (0.0%)	2 (3.3%)	2 (0.9%)
Year of surgery, *n* (%)					
2012–2016	0 (0.0%)	0 (0.0%)	0 (0.0%)	9 (14.8%)	9 (3.9%)
2017	16 (10.7%)	0 (0.0%)	16 (9.6%)	22 (36.1%)	38 (16.7%)
2018	25 (16.8%)	0 (0.0%)	25 (15.0%)	10 (16.4%)	35 (15.4%)
2019	45 (30.2%)	0 (0.0%)	45 (26.9%)	10 (16.4%)	55 (24.1%)
2020	25 (16.8%)	0 (0.0%)	25 (15.0%)	0 (0.0%)	25 (11.0%)
2021	38 (25.5%)	4 (22.2%)	42 (25.1%)	4 (6.6%)	46 (20.2%)
2022	0 (0.0%)	14 (77.8%)	14 (8.4%)	6 (9.8%)	20 (8.8%)
Length of surgery (minutes)	*n* = 146	*n* = 18	*n* = 164	*n* = 61	*n* = 225
Mean (SD)	19.4 (9.9)	21.1 (6.7)	19.5 (9.6)	43.9 (22.2)	26.1 (17.8)
Median (Min; Max)	17 (5; 51)*	20 (10; 30)*	17 (5; 51)**	33 (17; 104)**	20 (5; 104)
Anesthesia used					
Local	145 (97.3%)	18 (100.0%)	163 (97.6%)	58 (95.1%)	221 (96.9%)
Local and general	3 (2.0%)	0 (0.0%)	3 (1.8%)	0 (0.0%)	3 (1.3%)
General	1 (0.7%)	0 (0.0%)	1 (0.6%)	3 (4.9%)	4 (1.8%)
Implant type manufacturer	*n* = 149	*n* = 18	*n* = 167	*n* = 55	*n* = 222
Oticon Medical***	149 (100.0%)	18 (100.0%)	167 (100.0%)	48 (87.3%)	215 (96.8%)
Cochlear	0 (0.0%)	0 (0.0%)	0 (0.0%)	7 (12.7%)	7 (3.2%)
Implant length (mm)	*n* = 144	*n* = 18	*n* = 162	*n* = 60	*n* = 222
3	7 (4.9%)	0 (0.0%)	7 (4.3%)	6 (10.0%)	13 (5.9%)
4	137 (95.1%)	18 (100.0%)	155 (95.7%)	54 (90.0%)	209 (94.1%)
Abutment height (mm)	*n* = 117	*n* = 18	*n* = 135	*n* = 39	*n* = 174
6	0 (0.0%)	0 (0.0%)	0 (0.0%)	4 (10.2%)	4 (2.3%)
9	56 (47.9%)	6 (33.3%)	62 (45.9%)	22 (26.4%)	84 (48.3%)
12	57 (48.7%)	12 (66.7%)	69 (51.1%)	11 (28.2%)	80 (46.0%)
14	4 (3.4%)	0 (0.0%)	4 (3.0%)	2 (5.1%)	6 (3.4%)

* *p*-value MIPS vs. MONO = 0.48.

** *p*-value Minimally invasive vs. linear incision = <0.0001.

*** Ponto Wide implant and Ponto BHX implant.

The mean time of follow-up varied based on when the surgical techniques were introduced and implemented in clinical practice. The mean follow-up times were 3.09 years (SD = 2.26) and 1.65 (SD = 1.36) years for the linear incision group and minimally invasive group, respectively. The shortest mean follow-up time was represented by the MONO technique (0.128, SD = 0.139 years) as it was introduced in November 2021 (Table 3). Across all implantation techniques, there was a low number of observed intraoperative complications (2.6%) and even though fewer numbers were observed in the minimally invasive technique group (1.8%) than in the linear incision group (4.9%) there was no statistically significant difference detected (*p* = 0.39). More than half (53.5%) of the population experienced postoperative complications during their follow-up, with a significantly lower incidence in the minimally invasive group than in the linear incision group (49.1% vs. 65.6%, *p* = 0.039). Pain, scarring, and reported numbness did not, however, indicate statistical significance between the two surgical techniques. For the Holgers score, there was a statistical significance in the reported scores, demonstrating that the minimally invasive techniques are associated with fewer and less severe skin complications after surgery compared with the traditional linear incision technique. For example, 70% of the minimally invasive cases reported no skin reaction (Holgers = 0) during follow-up compared with 48% of the linear incision cases (*p* = 0.010) (Table 3).

**Table 3:** Complications and follow-up, presented per implant.

	MIPS (*n* = 149, 65.4%)	MONO (*n* = 18, 7.9%)	Minimal invasive (MIPS + MONO) (*n* = 167, 73.2%)	Linear incision (*n* = 61, 26.8%)	Total (*n* = 228)
Follow-up time (months)	*n* = 146	*n* = 18	*n* = 164	*n* = 57	*n* = 221
Mean (SD)	22.19 (16.27)	1.23 (1.30)	19.89 (16.69)	37.81 (27.12)	24.51 (21.34)
Median (Min; Max)	18.67 (0*; 57.9)	0.67 (0.20; 4.9)	15.48 (0*; 57.9)	36.93 (0.20; 124.53)	19.03 (0; 124.53)
Intra-operative complications, *n* (%)	*n* = 149	*n* = 18	*n* = 167	*n* = 61	*n* = 228
No	147 (98.7%)	17 (94.4%)	164 (98.2%)	58 (95.1%)	222 (97.4%)
Yes^a^	2 (1.3%)	1 (5.6%)	3 (1.8%)	3 (4.9%)	6 (2.6%)
Post-op complications, *n* (%)					
No	71 (47.7%)	14 (77.8%)	85 (50.9%)	21 (34.4%)	106 (46.5%)
Yes^b^	78 (52.3%)	4 (22.2%)	82 (49.1%)**	40 (65.6%)**	122 (53.5%)
Holgers score (0–4),^c^ *n* (%)	*n* = 146	*n* = 18	*n* = 164	*n* = 58	*n* = 222
0	98 (67.1%)	17 (94.4%)	115 (70.1%)	28 (48.3%)	143 (64.4%)
1	20 (13.7%)	0 (0.0%)	20 (12.2%)	12 (20.7%)	32 (14.4%)
2	7 (4.8%)	1 (5.6%)	8 (4.9%)	7 (12.1%)	15 (6.8%)
3	19 (13.0%)	0 (0.0%)	19 (11.6%)	8 (13.8%)	27 (12.2%)
4	2 (1.4%)	0 (0.0%)	2 (1.2%)***	3 (5.2%)***	5 (2.3%)
Holgers 0 or 1	118 (80.8%)	17 (94.4%)	135 (82.3%)	40 (69.0%)	175 (78.8%)
Holgers ≥2	28 (19.2%)	1 (5.6%)	29 (17.7%)	18 (31.0%)	47 (21.2%)
Scarring, *n* (%)	*n* = 146	*n* = 18	*n* = 164	*n* = 58	*n* = 222
No	132 (90.4%)	18 (100.0%)	150 (91.5%)	50 (86.2%)	200 (90.1%)
Yes	14 (9.6%)	0 (0.0%)	14 (8.5%)	8 (13.8%)	22 (9.9%)
Pain at last follow-up, *n* (%)	*n* = 146	*n* = 18	*n* = 164	*n* = 57	*n* = 221
No	132 (90.4%)	16 (88.9%)	148 (90.2%)	49 (86.0%)	197 (89.1%)
Yes	14 (9.6%)	2 (11.1%)	16 (9.8%)	8 (14.0%)	24 (10.9%)
Numbness at last follow-up, *n* (%)	*n* = 146	*n* = 18	*n* = 164	*n* = 57	*n* = 221
No	132 (90.4%)	18 (100.0%)	150 (91.5%)	53 (93.0%)	203 (91.9%)
Yes	14 (9.6%)	0 (0.0%)	14 (8.5%)	4 (7.0%)	18 (8.1%)
Implant lost, *n* (%)	*n* = 146	*n* = 18	*n* = 164	*n* = 57	*n* = 221
No	136 (93.2%)	18 (100.0%)	154 (93.9%)	51 (89.5%)	205 (92.8%)
Yes	10 (6.8%)^d^	0 (0.0%)	10 (6.1%)^d^	6 (10.5%)^e^	16 (7.2%)
Revision surgery, *n* (%)	*n* = 146	*n* = 18	*n* = 164	*n* = 58	*n* = 222
No	139 (95.2%)	18 (100.0%)	157 (95.7%)	53 (91.4%)	210 (94.6%)
Yes	7 (4.8%)	0 (0.0%)	7 (4.3%)	5 (8.6%)	12 (5.4%)
Abutment change, *n* (%)	*n* = 146	*n* = 18	*n* = 164	*n* = 58	*n* = 222
No	139 (95.2%)	18 (100.0%)	157 (95.7%)	47 (81.0%)	204 (91.9%)
Yes	7 (4.8%)	0 (0.0%)	7 (4.3%)	11 (19.0%)	18 (8.1%)

^a^ Intra-op complications included reports of implant instability, intensive bleeding, and prolonged surgery due to material issues.

^b^ Post-operative complications included reports of abutment instability, edema, granulation tissue, infection, and tinnitus.

^c^ Highest score reported during total follow-up period.

^d^ Spontaneous loss (*n* = 3), infection related (*n* = 3), elective removal (*n* = 2), trauma (*n* = 2),.

^e^ Spontaneous loss (*n* = 3), skin thickening (*n* = 2), elective removal (*n* = 1).

* 5 days.

** *p*-value Minimally invasive vs. linear incision = 0.039.

*** *p*-value Minimally invasive vs. linear incision = 0.010.

Implant survival for the whole cohort was 92.8%, with a reported implant loss rates of 6.1% and 10.5% for the minimally invasive and linear incision groups, respectively (*p* = 0.41). To date, there have been no reported implant losses in the group with implants installed using the MONO procedure (Table 3). Revision surgery was reported for 5.4% of the cases and abutment change was reported for approximately 8% of the cases with fewer observations seen in the minimally invasive group.

The dataset included a collection of audiological diagnoses and suggested that the majority of cases had a bilateral deficit with the most commonly reported codes bilateral conductive (43%) and bilateral mixed (33%) hearing loss. A unilateral etiology was observed in approximately 23% of the cases. The majority of cases (97%) were fitted with a Ponto sound processor (Oticon Medical AB, Askim, Sweden). The mean preoperative PTA4 air/bone on the implant side was reported as 77.9 (SD = 25.0)/41.1 (SD = 24.6) dB for the full population and the postoperative measurement was 33.6 (SD = 11.5) dB, indicating a significant improvement over time. The same pattern is seen, irrespective of surgical technique. See Table 4. Speech recognition is also improved from a mean of 48.3% (SD = 30.6) preoperatively to 92.6% (SD = 10.4%) postoperatively.

**Table 4:** Audiological etiology and descriptive measures, presented per implant.

	MIPS (*n* = 149)	MONO (*n* = 18)	Minimal invasive (MIPS + MONO) (*n* = 167)	Linear incision (*n* = 61)	Total (*n* = 228)
Audiological diagnosis^a^, *n* (%)					
Single sided deficit (H901; H904; H907)^a^	26 (17.4%)	2 (11.1%)	28 (16.8%)	6 (9.8%)	34 (14.9%)
H901: Unilateral conductive hearing loss	2 (1.3%)	0 (0.0%)	2 (1.2%)	3 (4.9%)	5 (2.2%)
H907; Unilateral mixed hearing loss	7 (4.7%)	1 (5.6%)	8 (4.8%)	5 (8.2%)	13 (5.7%)
H900: Bilateral conductive hearing loss	57 (38.3%)	9 (50.0%)	66 (39.5%)	31 (50.8%)	97 (42.5%)
H906: Bilateral mixed hearing loss	56 (37.6%)	6 (33.3%)	62 (37.1%)	13 (21.3%)	75 (32.9%)
Other^b^	1 (0.1%)	0 (0.0%)	1 (0.1%)	3 (4.9%)	4 (1.8%)
Sound processor manufacturer, *n* (%)	*n* = 144	*n* = 18	*n* = 162	*n* = 55	*n* = 217
Cochlear	0 (0.0%)	0 (0.0%)	0 (0.0%)	7 (12.7%)	7 (3.2%)
Oticon Medical	144**^c^** (100.0%)	18 (100.0%)**^d^**	162 (100.0%)	48 (87.3%)**^e^**	210 (96.8%)
Pre-op PTA (500/1 k/2 k/3 k/4 k), Implant side, BONE (dB)	*n* = 119	*n* = 11	*n* = 130	*n* = 35	*n* = 165
Mean (SD)	42.6 (24.1)	41.2 (22.5)	42.5 (23.9)	36.0 (27.0)	41.1 (24.6)
Median (Min; Max)	40 (0; 79)	34 (16; 77)	39.5 (0; 79)	33 (0; 76)	39 (0; 79)
Pre-op PTA (500/1 k/2 k/3 k/4 k), implant side, AIR (dB)	*n* = 119	*n* = 11	*n* = 130	*n* = 39	*n* = 169
Mean (SD)	78.9 (26.3)	71.3 (22.5)	78.2 (26.0)	76.7 (21.3)	77.9 (25.0)
Median (Min; Max)	73 (26; 130)	69 (42; 109)	72.5 (26; 130)	69 (36; 120)	71 (26; 130)
Post-op PTA (500/1 k/2 k/3 k/4 k), CL - Free Field Thresholds (dB)	*n* = 49	*n* = 2	*n* = 51	*n* = 29	*n* = 80
Mean (SD)	34.9 (12.1)	29.0 (8.5)	34.6 (12.0)	31.7 (10.4)	33.6 (11.5)
Median (Min; Max)	32 (15; 68)	29 (23; 35)	32 (15; 68)	30 (10; 57)	31 (10; 68)
Pre-op monosyllabic recognition (%)	*n* = 124	*n* = 18	*n* = 142	*n* = 44	*n* = 186
Mean (SD)	50.5 (29.2)	49.7 (29.5)	50.4 (29.2)	41.7 (34.4)	48.3 (30.6)
Median (Min; Max)	56 (0; 96)	56 (0; 84)	56 (0; 96)	44 (0; 100)	56 (0; 100)
Post-op monosyllabic recognition (%)	*n* = 129	*n* = 18	*n* = 147	*n* = 48	*n* = 195
Mean (SD)	92.4 (11.0)	89.8 (9.4)	92.1 (10.8)	94.1 (9.1)	92.6 (10.4)
Median (Min; Max)	96 (0; 100)	92 (60; 100)	96 (0; 100)	100 (56; 100)	96 (0; 100)

^a^ ICD-10-CM Diagnosis Codes (2022) used for coding. Generic/high-level code were used when specific subcategory was missing.

^b^ Single/few observations of general bilateral hearing deficit and other diagnoses.

^c^ Reported types; Ponto 3 (*n* = 2), Ponto 3 SuperPower (*n* = 107), Ponto Plus (*n* = 15), Ponto Plus Power (*n* = 20).

^d^ Reported types; Ponto 3 SuperPower (*n* = 18).

^e^ Reported types; Ponto 3 SuperPower (*n* = 19), Ponto Plus (*n* = 14), Ponto Plus Power (*n* = 14), Other (*n* = 1).

## 4. Discussion

### 4.1. Key results

The surgical techniques for installation of the BAHS have evolved significantly in recent years, particularly with the introduction of minimally invasive techniques such as MIPS and MONO, where the drilling procedure and installation are performed via a cannula inserted in a 4–5 mm circular skin incision (9, 10, 13). Here, we report the surgical and postoperative outcomes of using these techniques in a large number of patients in comparison with the conventional linear incision tissue preservation technique, demonstrating favorable results of MIPS and MONO in terms of surgery time and postoperative complications.

This study shows real-world data on the development of different surgical techniques when installing bone-anchored hearing implants and the associated trends in outcomes. As expected, the use of different surgical techniques follows availability on the market and subsequent introduction into clinical practice. As a result, there were no minimally invasive procedures reported between 2012 and 2016 and in the latest (full) years (2019–2021), 91% of BAHS implantations were performed with minimally invasive techniques (Table 2). It should be noted that the latest development in minimally invasive techniques, MONO, was introduced in late 2021 and there is a limited follow-up time and cases (*n* = 18) associated with this method. Further studies are warranted to determine the clinical outcome associated with using the MONO procedure.

Overall, the minimally invasive technique offers less complexity and very few intraoperative complications. In agreement with other reports utilizing MIPS, the surgery duration was significantly shorter, with 20 vs. 44 min in favor of minimal invasive techniques, without increasing complication rates (6, 17, 21). In addition, there was a trend of fewer intraoperative complications reported in the group of minimally invasive surgeries (1.8% vs. 4.9%), which supports the use of this surgical approach in an in-office setting, out of the main operating room, as suggested by King et al. 2022 (22). This could potentially also provide an added cost-benefit as described by Sardiwalla et al., 2017 and Strijbos et al., (23, 24).

The number of postoperative complications seems to generally decline over the years as the surgical techniques, implant designs and postoperative care have improved. The occurrence of any postoperative complication was more commonly reported in the linear incision group (66%) than in the minimally invasive group (49%) but when comparing specific subcategories such as pain, scarring, and numbness no reliable differences were detected. However, Holgers scores indicate that there are fewer and less severe skin complications after minimally invasive procedures, which is in line with previously reported findings in the literature (4, 17, 18, 25). The reported Holgers scores were in accordance with those previously published by Amaral et al., 2020 (16) but slightly higher than those reported in the systematic review by Lagerkvist et al., 2020 (5) which is possibly explained by the unique study setting. The study data included a wide range of ages and although the total sample only included 8% patients below the age of 12 years it is possible that this group is over-represented among the complication rates as previously reported by Bezdjian et al. 2018 (26).

A randomized, controlled, multicenter study comparing MIPS with linear incision with tissue preservation reported an elevated implant loss rate for the MIPS group and proposed several factors possibly contributing to this, including (i) learning curve and training (ii) soft tissue entrapment in osteotomy and (iii) the lack of direct access and full visibility to the surgical site causing insufficient irrigation and angulated insertion of the implant (6). In the present study however, the implant survival was comparable between the MIPS and linear incision, and both were comparable to data previously published data for systems BAHS with the wide diameter implant (Table 3) (27). Furthermore, the drill system has been slightly updated with the aim of reducing the risk of thermal damage to the bone during osteotomy preparation (17).

This extensive follow-up further suggests that the long-term implant survival rate could be expected to be approximately 93% for bone-anchored hearing implants, when used as intended in clinical practice. Data from audiological measurements support that there is a general benefit of undergoing surgery with improvements in both pure tone average and speech recognition measurements. The data do not support any differences in implant survival or audiological outcomes between minimally invasive or linear incision techniques.

### 4.2. Limitations

There are many limitations associated with the retrospective nature of this study. For example, causality relationships should be interpreted with care as there are many potential confounding factors that could explain the observed variations. For example, there is always a chance that the differences we see in surgical techniques are a general effect from improvements over time in the operational room setting, patient candidacy, and/or clinical practice routines and not only attributed to the selected surgical method. Nevertheless, the observed differences are still present, and the difference in for example surgery duration suggests a significant reduction in time spent, which proposes some relevance of the results despite the limitations. In general, heterogeneous real-world data always risk masking true benefits associated with an intervention but again, we believe that the results presented in this study are strengthened by the number of observations (228 implant installations in 200 patients over 10 years), suggesting that a clinically relevant difference should stand out despite confounding factors.

It is easy to assume that the choice of surgical technique follows the complexity of a case and that might be true for the individual patient. However, in the current cohort, the type of method mainly followed the timepoint of surgery, indicating that it mainly represents the dominating clinical practice at the time of surgery. For the same reason, we observed different follow-up times for the different techniques. The linear incision technique was originally adopted as the first-choice method and was therefore exclusively represented in the early collection period between 2012 and 2016 and then gradually replaced by the minimally invasive techniques. As a result, longer follow-up times are reported in the linear incision group (3.1 ± 2.3 years) than in the minimally invasive group (1.7 ± 1.3 years). This difference could potentially explain the observed changes in complication rates, but it should be noted that complication rates are not cumulatively collected but instead reported as occurrence or not during the follow-up period. For example, the Holgers score is reported as the highest score during the full follow-up period, irrespective of when it occurred. It is also assumed that most surgery-related complications occur during the early follow-up period. If we exclude the shortest follow-up times in the comparison in postoperative complication rates, i.e., exclude the MONO cases and compare MIPS to linear incision cases only, we still see a statistically significant difference in the occurrence of postoperative complication rates of 52% vs. 66%, respectively (*p* = 0.033).

### 4.3. Interpretation and generalizability

We suggest that the current study results should be seen as a measurement of effectiveness when adopting minimally invasive techniques into clinical practice for bone-anchored hearing implants and that the results have a high degree of generalizability. This, as there is a low risk of selection biases, and the data represent an unusually large sample of real-world observations and a total population with wide variability in age groups, hearing loss etiology, and audiological diagnoses, collected over a total period of 10 years.

We suggest some care in interpreting findings from the audiological measurements as there are some missing values and inconsistencies in the methods. These data should only be used to provide an indication of general improvement and clinical benefits of the treatment of a bone-anchored hearing implant.

There are some missing data on abutment and implant type, but the vast majority of data represent one legal manufacturer as the minimally invasive technique is only offered by them. The data is too limited and not suitable to draw conclusions on possible product differences between manufacturers.

## 5. Conclusions

Adoption of a minimally invasive surgical technique for installation of bone-anchored hearing implants can reduce surgical complexity without compromising safety aspects or clinical benefits. Shorter surgery time and fewer complications, especially those associated with the skin and wound healing, could be expected when going from a linear incision to a minimally invasive technique. The findings in the current study support using the minimally invasive techniques in an in-office setting, outside the main operating room.

## Data Availability

The original contributions presented in the study are included in the article, further inquiries can be directed to the corresponding author.
